# Unfertilized *Xenopus* Eggs Die by Bad-Dependent Apoptosis under the Control of Cdk1 and JNK

**DOI:** 10.1371/journal.pone.0023672

**Published:** 2011-08-16

**Authors:** David Du Pasquier, Aude Dupré, Catherine Jessus

**Affiliations:** 1 CNRS, UMR 7622-Biologie du Développement, Paris, France; 2 Université Pierre et Marie Curie-Paris 6, UMR 7622-Biologie du Développement, Paris, France; Ecole Normale Supérieure de Lyon, France

## Abstract

Ovulated eggs possess maternal apoptotic execution machinery that is inhibited for a limited time. The fertilized eggs switch off this time bomb whereas aged unfertilized eggs and parthenogenetically activated eggs fail to stop the timer and die. To investigate the nature of the molecular clock that triggers the egg decision of committing suicide, we introduce here *Xenopus* eggs as an *in vivo* system for studying the death of unfertilized eggs. We report that after ovulation, a number of eggs remains in the female body where they die by apoptosis. Similarly, ovulated unfertilized eggs recovered in the external medium die within 72 h. We showed that the death process depends on both cytochrome c release and caspase activation. The apoptotic machinery is turned on during meiotic maturation, before fertilization. The death pathway is independent of ERK but relies on activating Bad phosphorylation through the control of both kinases Cdk1 and JNK. In conclusion, the default fate of an unfertilized *Xenopus* egg is to die by a mitochondrial dependent apoptosis activated during meiotic maturation.

## Introduction

Apoptosis is critically important in various developmental processes where it re-equilibrates the overproduction of cells occuring in several tissues [1]. In the adult, it is also central in maintaining homeostasis and remodeling. Apoptosis is a widespread event in oogenesis where it assumes all of these functions. One apoptotic event regards ovulated matured eggs. Eggs become capable of being fertilized after having completed oocyte meiotic maturation, a process that involves a partial or complete passage through meiotic divisions and occurs at time of ovulation. In vertebrates ovaries, oocytes are arrested in prophase during the first meiotic division (prophase I). Meiotic maturation depends on the activation of the master regulator of M-phase, MPF (M-phase promoting factor, or Cdk1 kinase) that promotes the transition from the prophase I arrest to the metaphase arrest of the second meiotic division [2]. From this point on, ovulated cells are called eggs and remain arrested in metaphase II because of high levels of CSF (cytostatic factor) activity that stabilizes MPF until fertilization [3]. Mos, a MEK kinase, is an integral component of CSF activity and is responsible for activating the MAP kinase pathway in maturing oocytes and eggs. It has been shown in several species that ovulated eggs possess a maternal machinery of apoptotic execution that is inhibited for limited time. The fertilized eggs can switch off this time bomb whereas aged unfertilized eggs and parthenogenetically activated eggs fail to stop the timer and commit suicide [4]–[12]. Indeed, unfertilized eggs may suffer from aging, leading to abortive or abnormal development, explaining the biological importance of this post-ovulatory apoptotic process. There is therefore great interest in understanding unfertilized egg apoptosis in relation to failed conception and birth defects that dramatically increase with post-ovulatory age.

However, the fate of unfertilized eggs has received little attention, except for a limited number of studies on mammals and starfish [4]–[12]. Not only are starfish evolutionarily distant from vertebrates, but details of their eggs' maturation, shedding and fertilization are also very different, making comparisons difficult. The studies conducted in mammals are complicated by the difficulty in obtaining high oocyte amounts and the requirement to carry out the experimentation *in vitro* while the eggs age and die within the female reproductive tract. We therefore sought to introduce *Xenopus* eggs as an *in vivo* system for studying the death of unfertilized eggs. This model offers many advantages that are lacking in others: it is a vertebrate system physiologically closer from mammals than any invertebrate, oocyte meiotic maturation and aging occur externally, the model system is transcription-independent, and the high number of large oocytes are amenable to *in vivo* experimental manipulation.

We observed that after stimulated ovulation, a number of *Xenopus* eggs failed to be laid and remained in the female body where they die by apoptosis, a situation similar to the one described in mammals. Thus, the default fate of an unfertilized *Xenopus* egg is to die by apoptosis. In order for a fertilized egg to develop properly, fertilization must occur before the maternal apoptosis program is irreversibly activated. To understand normal development, it is therefore important to know how eggs undergo apoptosis. In most cells, the key executioners of apoptosis are members of a protease family known as the caspases that cleave cellular substrates and disrupt cell integrity [13]. In many cell types, apoptosis depends on a mitochondrial pathway based on the release of cytochrome c (Cyt c) from the intermembrane space of the mitochondria to the cytoplasm. Cyt c release is regulated by the Bcl-2 family of proteins that encompasses proapoptotic members, such as Bax and Bad, and antiapoptotic members, such as Bcl-xL or Mcl-1 [14], [15]. Proapoptotic proteins, as Bax, allow proteins in the mitochondrial intermembrane space, such as Cyt c, to escape into the cytosol [15]. Once released into the cytosol, Cyt c forms a multimeric complex with Apaf-1 and renders Apaf-1 competent to activate procaspase 9, which subsequently cleaves and activates the effector caspase 3 [16]. Another death pathway involves the activation of death receptors by death ligands and the activation of caspase 8 which in turn will activate the executioner caspase 3 [16]. Caspase 3 represents the workhorse of execution, resulting in specific morphological features including cell shrinkage, chromatin condensation, formation of cytoplasmic blebs and apoptotic bodies and finally phagocytosis of the apoptotic bodies [17]. In this study, we demonstrate that *Xenopus* eggs synchronously die within 72 h by mitochondrial and caspase-dependent apoptosis. The molecular clock that ultimately triggers the decision of committing death in the absence of fertilization relies on the phosphorylation of Bad, an event depending on the activation of both kinases Cdk1 and JNK during meiotic maturation.

## Results

### Ovulated eggs that are not laid, die by apoptosis in the genital tract

This study used *Xenopus* eggs as an in vivo system for studying unfertilized egg apoptosis. Oocytes present in the ovary are blocked in prophase of the first meiotic division. Upon hormonal stimulation of the female, oocytes resume meiotic maturation and are ovulated. The nuclear envelope breaks down (GVBD for germinal vesicle breakdown), the first meiotic division occurs, and the oocyte, now called egg, arrests at the metaphase of the second meiotic division. These matured eggs are characterized by a white depigmented spot at the animal pole. The maturing ovulated eggs are released into the coelom and oriented to the opening of the oviduct. Through their passage in the oviduct, they become surrounded by a mucoid jelly-coat secreted by oviduct glands. The end of the oviduct broadens to form the uterus and eggs are then transferred to the cloaca, before deposition in external medium.

**Figure 1:**
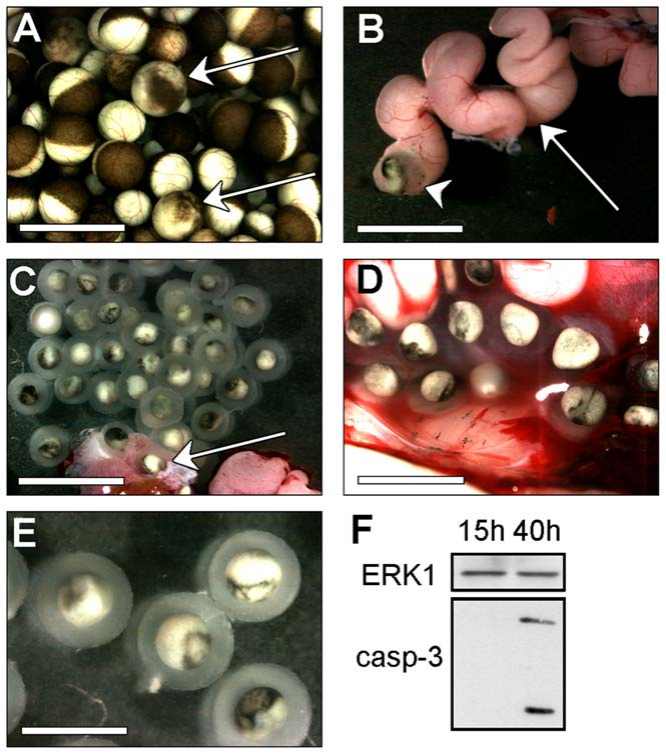
Unlaid matured eggs, die by apoptosis in the female frog body. *Xenopus* females were induced to lay by hCG injection. Laying started 15 h after hormonal stimulation and was completed 24 h after. Forty hours after injection, females were dissected and dying eggs with altered pigmentation could be observed in the animal body: trapped in the ovary (A, arrows indicate dying eggs, bar = 2.4 mm), in the oviduct (B, arrow-head indicates a dying egg at the exit of the oviduct, arrow indicates a dying egg in the oviduct visible through the wall, bar = 3 mm), in the uterus (C, arrow indicates the opened uterus, bar = 5 mm) or in the cloaca (D, bar = 3 mm). Representative dying eggs are shown in (E, bar = 2.4 mm). (F) Caspase 3 activation was measured by immunoblot using an antibody against the cleaved active form of caspase 3 (casp-3) in eggs laid 15 h after hCG injection and dying eggs trapped in the genital tract 40 h after hCG injection. The ERK1 protein was immunoblotted as a loading control.

We first investigated whether all ovulated eggs are laid, as expected from an oviparous species, or whether some of them are retained in the reproductive tract of the female. Females were stimulated by hCG and started to lay 15 h after hormonal stimulation. After 24 h, laying was completed. We dissected the females 40 h after hCG treatment. Surprisingly, fully-grown eggs with an altered pigmentation were recovered in the ovary, among healthy prophase oocytes (Figure 1A). In contrast, the observation of the ovaries of a control non-stimulated female did not reveal any abnormalities in the pigmentation of oocytes (not shown). Identical abnormally pigmented eggs were recovered in the oviduct, in the uterus and in the cloaca, all of them surrounded by a jelly-coat (Figure 1B–E). This observation indicates that these eggs have been ovulated and normally transferred in the genital ducts but were not laid. They represent a small population (50 to 200 per animal) considering the amount of laid eggs (2 to 5 thousands). Given the altered distribution of the melanosomes of the animal hemisphere that covers an irregular and reduced area, we hypothesized that these eggs could be dying by apoptosis. Almost all apoptotic pathways converge to the activation of caspase 3 as final executioner of the death program [18]. We therefore analyzed the presence of the activated cleaved form of caspase 3 by immunoblot as a marker of apoptosis (Figure 1F). As shown in Figure 1F, ovulated eggs retained in the same female body, contain the active form of caspase 3, as revealed by western blotting with an antibody recognizing specifically the active cleaved forms of caspase 3. In contrast, freshly laid eggs from the same female 15 h after hCG stimulation do not exhibit activated caspase 3 (Figure 1F). The protein kinase ERK1 was used as a loading control, as the protein is abundantly expressed in oocytes and eggs and its level is not affected by apoptosis (Figure 1F). Therefore, although *Xenopus* is oviparous with external fertilization, a significant amount of ovulated eggs are not laid and these cells are inactivated inside the female body by apoptosis. This situation is quite similar to the one described in mammals [7], [12].

### *Xenopus* unfertilized matured eggs undergo apoptotic cell death

**Figure 2:**
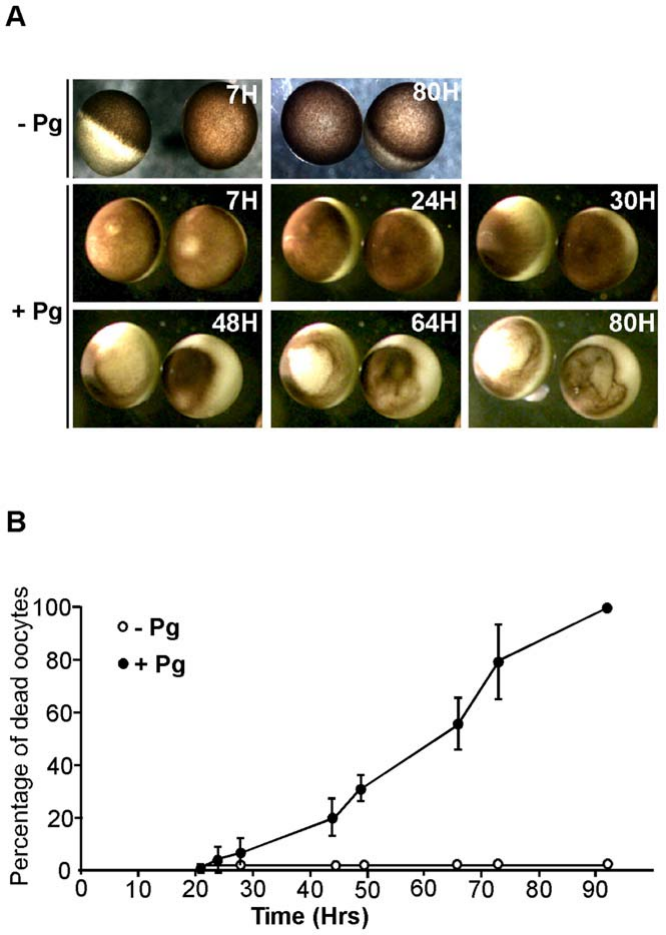
Time-course of morphological changes characterizing the spontaneous death of progesterone matured oocytes. Fully-grown prophase oocytes were isolated from the ovaries and incubated in the presence (+Pg) or in the absence (−Pg) of 1 µM progesterone. (A) Oocytes originating from one female were photographed at the indicated times. (B) Time-course of death of either prophase-blocked oocytes (−Pg, white circles) or progesterone-matured oocytes (+Pg, black circles) from eight different females. Oocytes were considered as dead when their external morphology was similar to the one occurring at 48 h in panel A.

To study the molecular mechanisms underlaying the execution of this apoptosis event, we took advantage of the frog oviparity where oocyte maturation and aging of the majority of the eggs occur externally. Prophase fully-grown oocytes were isolated from the ovary and incubated in the presence of progesterone, the hormone responsible on re-entry into meiosis at time of ovulation [2]. The oocytes underwent meiotic maturation within 7 h and exhibited a white spot at the animal pole characterizing the metaphase II-arrest (Figure 2A). The incubation of the metaphase II-arrested eggs was then extended to analyze whether these cells adopt the same external morphology as those recovered in the female body (Figure 1). One representative experiment is illustrated in Figure 2A. Starting 24 h, the white spot disappeared and the pigmented area started to shrink. After 30 h, the contraction of the pigment progressed and starting 48 h, the melanosomes were gathered in a restricted area, either compact or surrounding a white depigmented large area (Figure 2A). An identical phenotype was observed in unfertilized laid eggs surrounded by a jelly-coat, and incubated in the external medium for two days after the deposition by the female (data not shown). These pigment rearrangements are strikingly different to those observed after parthenogenetic activation [19]. Osmotic stress is also known to induce pigment changes: eggs become white and swollen, and the cytoplasm leaks out. We never observed any cytoplasmic leakage but rather a cytoplasmic contraction. Figure 2B represents a quantitative analysis of experiments performed with 8 females. In all cases, the first pigment rearrangements started 24 h after meiosis induction by progesterone. Then, the time-course of the pigment rearrangement was dependent on the female and on the external temperature (being accelerated at 24°C in comparison to 18°C). In contrast, prophase-blocked oocytes can be routinely maintained in culture for more than four days without exhibiting any alteration of their external morphology (Figure 2A). Therefore, the signal transduction pathway leading to cell death is likely to be inactive in prophase-blocked oocytes and the activation of the death inducer occurs during meiotic maturation.

**Figure 3:**
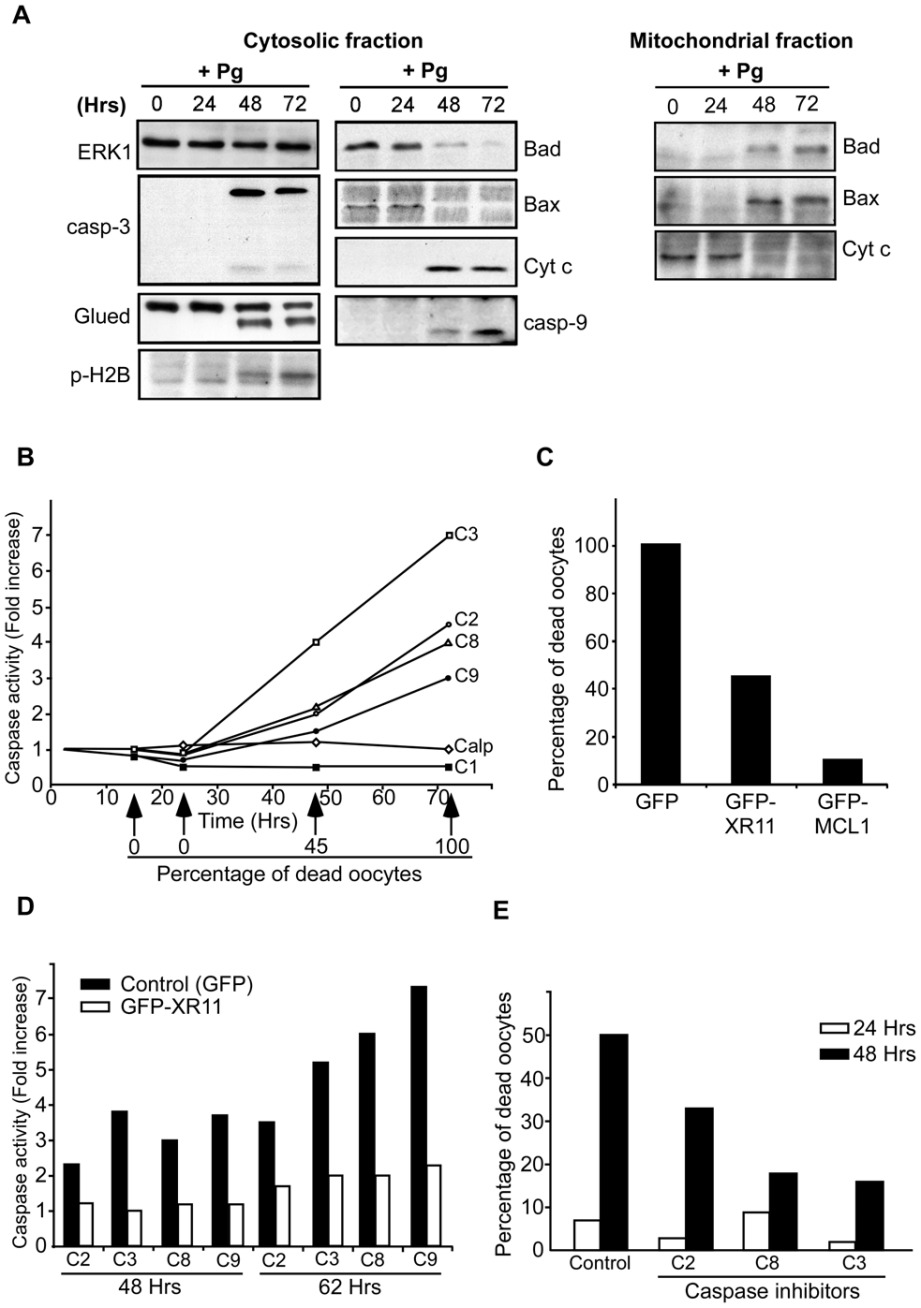
Mitochondrial- and caspase dependent apoptosis is responsible for unfertilized egg death. Fully-grown prophase oocytes were isolated from the ovaries and stimulated by 1 µM progesterone. (A) Western blot analysis of cytosolic and mitochondrial fractions of unfertilized eggs was performed at various times following progesterone addition (+Pg) with antibodies against ERK1, the cleaved form of caspase 3 (casp-3), p150Glued, the Ser14-phosphorylated form of histone H2B (p-H2B), Bad, Bax, Cyt c and the cleaved form of caspase 9 (casp-9). (B) Caspase and calpain activities of unfertilized eggs were assayed at various times after progesterone stimulation with the following substrates: Suc-LY-AMC (calpain, Calp, white diamonds), Ac-YVAD-AMC (caspase 1, C1, dark squares), Z-VDVAD-AFC (caspase 2, C2, white circles), DEVD-AMC (caspase 3, C3, white squares), Z-IETD-AFC (caspase 8, C8, white triangles) and Ac-LEHD-AFC (caspase 9, C9, black circles). In parallel, the percentage of dead eggs was followed using morphological criteria. (C) Oocytes were microinjected with mRNAs encoding either GFP, GFP-XR11 or GFP-Mcl-1 fusion proteins and then incubated in the presence of 1 µM progesterone. The percentage of dead eggs was estimated 70 h later using morphological criteria. (D) Oocytes were microinjected with mRNAs encoding either GFP (control, black columns) or GFP-XR11 fusion protein (white columns). They were then incubated in the presence of 1 µM progesterone. Caspase 2 (C2), caspase 3 (C3), caspase 8 (C8) and caspase 9 (C9) activities were assayed 48 and 62 h later as described in (B). (E) Oocytes were microinjected with various caspase inhibitors: VDVAD-FMK (caspase 2, C2), DEVD-FMK (caspase 3, C3) or IETD-FMK (caspase 8, C8). Progesterone was added and the percentage of dead eggs was estimated 24 h (white columns) and 48 h (black columns) later after morphological criteria. All data shown in these panels are representative experiments repeated on oocytes and eggs from at least three different females.

The morphological changes affecting matured eggs in the external medium are similar to those observed in the ovulated eggs retained in the female body that exhibit active caspase 3. We therefore analyzed whether this altered phenotype corresponds to death by apoptosis. We first ascertained the activity of caspase 3 by western blotting. ERK1 protein was used as a loading control. The effector caspase 3 was activated 48 h after progesterone stimulation (Figure 3A). To confirm the involvement of caspase-dependent cleavages in unfertilized *Xenopus* eggs, we analyzed by western blot the dynactin subunit p150Glued, that is cleaved by caspases both in apoptotic *Xenopus* egg extracts and during apoptosis in human and rat cell lines [20]. As shown in Figure 3A, the monoclonal antibody detected a cleavage product in 48 h-incubated eggs, 10–20 kD smaller than the native molecule, a pattern identical to the one reported in apoptotic egg extracts. Another hallmark of apoptosis is provided by phosphorylation of histone H2B at Ser14 that correlates with cells undergoing apoptosis in vertebrates [21]. An antibody directed against Ser14-phosphorylated H2B revealed that histone H2B is phosphorylated on Ser14 at time of caspase 3 activation and p150Glued cleavage in unfertilized eggs (Figure 3A).

### Contribution of mitochondria and various caspases to the unfertilized egg apoptotic process

We then addressed the question of the implication of a mitochondrial phase in the processing of egg apoptosis. As a first marker of mitochondrial implication, we elected to monitor efflux of Cyt c from the mitochondrial intermembrane space. At various time points after initiating meiotic maturation by progesterone, cytosolic fractions were separated from the organelle fraction. Both fractions were then assayed for the presence of Cyt c by immunoblotting (Figure 3A). By 48 h, the mitochondria had released significant quantities of Cyt c. This was correlated with activation of caspase 9, as ascertained by western blot with an antibody specific of its active form and not recognizing the inactive uncleaved procaspase 9 (Figure 3A). As last markers of the involvement of a mitochondrial phase in the process, we investigated the regulation of Bad and Bax. Bad and Bax were detected in the egg cytosolic fraction during the period preceding caspases 3 and 9 activation, but disappeared from the cytosol by 48 h-incubation and were then recovered in the organelle fraction (Figure 3A).

Caspases are functionally grouped into initiator caspases (as caspases 8, 2 and 9) and effector caspases, as caspase 3. Other proteases, including calpain, have been found to be involved in a few cases of cell death. Finally, some caspases, as caspase 1, are associated primarily with cytokine activation and not directly with apoptosis [13]. We directly monitored the activity of these different classes of proteases in unfertilized eggs undergoing spontaneous apoptotic death by using specific cleavage substrates. As expected, calpain and caspase 1 were not activated during the process whereas caspases 3 and 9 underwent activation by 48 h after progesterone addition (Figure 3B), in agreement with the molecular analysis illustrated in Figure 3A. Interestingly, two initiator caspases, caspases 8 and 2, were also activated with a similar time-course (Figure 3B).

We then assessed the implication of anti-apoptotic members from the Bcl-2 family in the execution of the apoptotic process. Oocytes were microinjected with mRNA encoding tag-versions of either XR11 (the *Xenopus* homolog of Bcl-X_L_) or *Xenopus* Mcl-1, then stimulated by progesterone, and apoptosis was analyzed 70 h later by observing egg morphology. Overexpression of XR11 inhibited the apoptotic process by 50% whereas apoptosis was almost totally prevented by Mcl-1 (Figure 3C). In the following experiments, XR11 was used to prevent egg death since the physiological effects of the protein as a caspase inhibitor had already been characterized in *Xenopus* [22], [23] and since we noticed that exogenous Mcl-1 is unstable when expressed in the whole oocyte, as similarly observed in egg extracts [24]. Activity of caspases 3, 9, 2 and 8 was assayed in eggs where apoptosis is impaired by XR11 overexpression. The activity of all these caspases was strongly reduced in XR11 overexpressing cells when compared to control GFP-injected eggs (Figure 3D). These results show that a mitochondrial phase and the downstream activation of caspases 9 and 3 are required for the execution of the egg death. These data also suggest that activation of caspases 8 and 2 depends on this mitochondrial phase and probably do not play the upstream role of initiator caspases. To assess the implication of these caspases during egg death, oocytes were microinjected with various caspase inhibitors, then stimulated by progesterone and apoptosis was analyzed at 24 and 48 h. As expected, the specific caspase 3 inhibitor efficiently prevented apoptosis while the caspase 2 inhibitor had a moderate effect (Figure 3E). The caspase 8 inhibitor strongly delayed the process but did not block it (Figure 3E). We concluded that the death process involves all these caspase activities. Even though the initiator caspases 2 and 8 participate to the death process, they are recruited downstream of the mitochondrial phase.

### The two major meiotic kinases, Cdk1-cyclin B and ERK1, are inactivated when unfertilized eggs enter apoptosis

**Figure 4:**
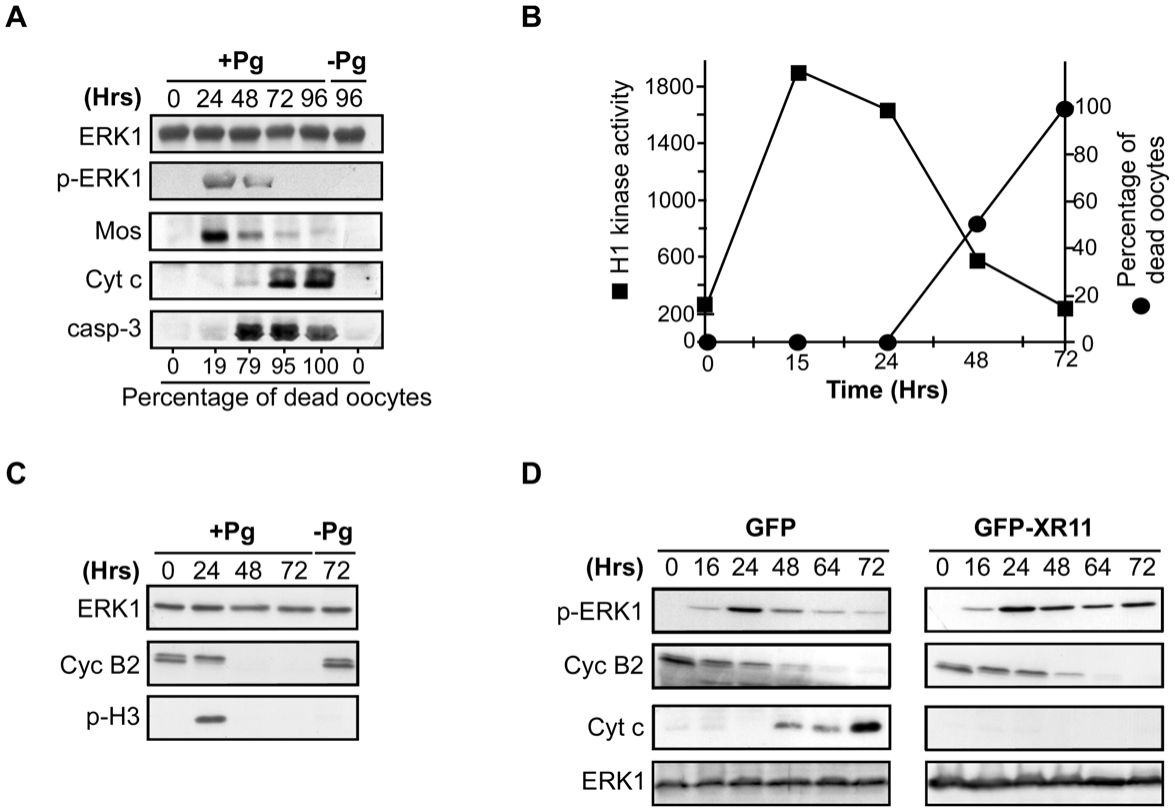
ERK1 is inactivated in response to apoptosis whereas Cdk1 inactivation occurs during egg aging, independently of apoptosis. Fully-grown prophase oocytes were isolated from the ovaries, stimulated by 1 µM progesterone (+Pg, time 0) or not (−Pg) and then incubated for various times. Three females were used, one in (A), another in (B) and (C) and one in (D). (A) Western blot analysis of cytosolic fractions of unfertilized eggs was performed at various times after progesterone stimulation with antibodies against ERK1, the active phosphorylated form of ERK1 (p-ERK1), Mos, Cyt c and active caspase 3 (casp-3). The percentage of eggs undergoing apoptosis after morphological analysis is indicated at the bottom, the incubation times in hours (Hrs) are indicated at the top. (B) H1 kinase activity of Cdk1 and apoptotic death were assayed in eggs at various times (in Hours, Hrs) after progesterone stimulation. (C) Western blot analysis of cytosolic fractions of unfertilized eggs was performed at various times after progesterone stimulation (+Pg) with antibodies against ERK1, cyclin B2 (Cyc B2) and the Ser10-phosphorylated form of histone H3 (p-H3). The incubation times in hours (Hrs) are indicated at the top. (D) Oocytes were microinjected with either GFP or GFP-XR11 mRNA and then stimulated by progesterone (time 0). Western blot analysis of cytosolic fractions of unfertilized eggs was performed at various times after progesterone stimulation with antibodies against ERK1, the active phosphorylated form of ERK1 (p-ERK1), cyclin B2 (Cyc B2) and Cyt c. The incubation times in hours (Hrs) are indicated at the top.

Meiotic maturation depends on the interplayed activities of two kinases, Cdk1-cyclin B and ERK1 [2]. The metaphase II-arrest is ensured by a high activity level of Cdk1, due to the stability of cyclin B that is under the indirect control of Mos/MEK/ERK1 cascade. Fertilization provokes degradation of cyclin B within 10 minutes and meiotic exit whereas the Mos pathway is inactivated one hour later [3]. The activity of both Cdk1 and ERK1 had never been investigated during aging of *Xenopus* unfertilized eggs. Metaphase II-arrested eggs were incubated for 96 h and the activity of the Mos pathway was analyzed by western blot following two criteria: the phosphorylation level of ERK1, which correlates with its activation, and the presence of Mos. Figure 4A shows that ERK1 activity was high at 24 h, then decreased at 48 h and was undetectable at 72 h, in correlation with the expression pattern of Mos that started to be degraded at 48 h. In this experiment, the apoptotic death was initiated at 24 h and, at 48 h, 79% of the eggs had undergone apoptosis, as ascertained by morphological changes and as confirmed by the detection of soluble Cyt c and active caspase 3 (Figure 4A). These observations indicate that apoptosis starts before the inactivation of the Mos pathway. Cdk1-cyclin B activity was analyzed in the same type of experiments, by assaying H1 kinase activity, the expression level of cyclin B2 and the phosphorylation level of Ser10 of histone H3, a usual marker of M-phase [25]. Cdk1 activity started to decline after 24 h of incubation and was reduced by 78% at 48 h, in correlation with Cyclin B2 degradation and Ser10 dephosphorylation of Histone H3 (Figure 4B–C). In this experiment, eggs did not exhibit any morphological characteristics of apoptosis at 24 h while 50% died at 48 h (Figure 4B). These results reveal that Cdk1-cyclin B inactivates slightly before the initiation of the apoptotic process.

We next investigated whether inactivation of ERK1 and Cdk1-cyclin B is a consequence either of apoptosis or aging of the egg. To address this question, apoptosis was prevented by injecting mRNA encoding XR11 and then both ERK1 and Cdk1 activities were analyzed during egg aging. In this experiment, XR11 inhibited egg death, as ascertained by the morphological changes affecting the eggs and the absence of Cyt c release (Figure 4D). Interestingly, ERK1 activity remained stable for 72 h during aging of eggs whose apoptotic death was prevented by XR11 overexpression whereas it decreased around 48 h, at the time of apoptosis, in control eggs (Figure 4D). This result strongly suggests that ERK1 inactivation is a downstream event depending on the execution of the mitochondrial phase of apoptosis. To confirm this conclusion, metaphase II-arrested eggs were incubated in the presence of U0126, a pharmacological inhibitor of MEK [26]. Under these conditions, ERK1 was inactivated within 2 h, but the time-course of apoptosis was not affected (not shown), supporting the conclusion that ERK1 inactivation is not a requirement for apoptosis induction. In contrast, cyclin B2 was degraded with a similar time-course in aged eggs undergoing or not apoptotic death (Figure 4D). Therefore, inactivation of Cdk1 is a spontaneous process occuring during egg aging, independently of apoptosis induction.

### Both Cdk1 and JNK control apoptosis of unfertilized eggs

**Figure 5:**
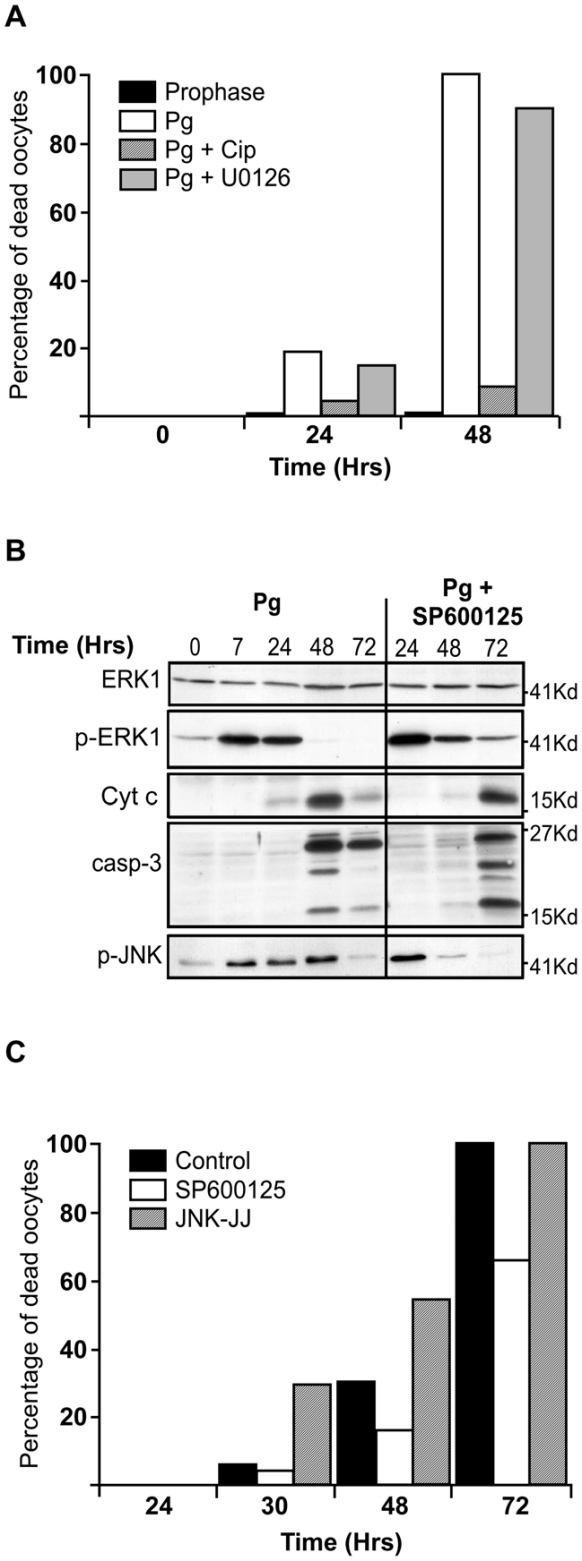
Egg apoptosis involves JNK and Cdk1 activities. (A) Prophase oocytes were either incubated with a MEK inhibitor (U0126, grey columns) or injected with the p21^Cip1^ protein (Cip, crosshatched column) and then stimulated (Pg, white column) or not (prophase, black column) with progesterone. Egg death was monitored by following external egg morphology at the indicated times (in hours, Hrs) after progesterone addition (+Pg). (B) Prophase oocytes were stimulated with progesterone (Pg, time 0). Seven hours later, metaphase II-arrested eggs were incubated with or without the JNK inhibitor, SP600125. Egg proteins were analysed at the indicated times by immunoblot with antibodies against ERK1, the active phosphorylated form of ERK1 (p-ERK1), Cyt c, active caspase 3 (casp-3) and the active phosphorylated form of JNK (p-JNK). (C) Oocytes were induced to mature as in (B) and were then incubated with (white column) or without (black column) the JNK inhibitor, SP600125, or injected with mRNA encoding a constitutive active form of JNK (JNK-JJ, crosshatch columns). Egg death was monitored by following external egg morphology for all these three different conditions at the indicated times.

To ascertain that Cdk1 activity is a pre-requisite for the emergence of apoptotic cell death in unfertilized eggs, Cdk1 activation induced by progesterone was prevented by the microinjection of p21^Cip1^, an inhibitor of Cdk1 (the only Cdk expressed in the oocyte) [27], into prophase oocytes (Figure 5A). Prophase oocytes were also incubated in the presence of U0126, a MEK inhibitor that leads to the inhibition of ERK activation [26], [28], [29] (Figure 5A). Apoptotic cell death was followed 24 h and 48 h after progesterone addition. As previously reported [27], [28], p21^Cip1^ completely blocked meiotic maturation whereas the MEK inhibitor delayed the process (not shown). Absence of ERK activity did not prevent the induction and the completion of apoptosis (Figure 5A). In contrast, preventing Cdk1 activation completely inhibited the induction of apoptosis (Figure 5A). Altogether, these results clearly establish that the induction of apoptosis during meiotic maturation depends on Cdk1 activity and not on ERK1 activation.

The Jun N-terminal kinases (JNKs) are a subfamily of MAP kinases that play critical roles in stress responses and apoptosis [30]. *Xenopus* oocytes stably express JNK whose activity increases abruptly just before GVBD in response to progesterone and stays active thereafter [31]. To analyze the role of JNK in the induction of apoptosis of unfertilized eggs, eggs were first stimulated by progesterone and after completion of meiotic maturation, were treated with a pharmacological inhibitor of JNK, SP600125 [32]. The spontaneous process of apoptosis was then analyzed by following morphological changes of eggs, Cyt c release and activation of caspase 3. As previously reported [31], [33], JNK was activated during meiotic maturation, as ascertained by the immunoblot detection of its active phosphorylated form (Figure 5B). JNK remained active for at least 48 h, when the apoptotic process had already started and ERK1 had been already inactivated. JNK was then inactivated after 72 h of incubation, when 100% of eggs completed apoptosis (Figure 5B). As expected, the SP600125 inhibitor inhibited JNK activity that was returned to a basal level by 48 h of incubation (Figure 5B). Interestingly, the JNK inhibitor delayed the initiation of the apoptotic process, and as a consequence, ERK1 inactivation (Figure 5B–C). To ascertain the implication of JNK in the apoptosis induction, the reverse experiment was performed by overexpressing an active form of JNK. Oocytes were injected with mRNA encoding a constitutive form of JNK, JNK-JJ [34] and were then stimulated with progesterone. Pigmental rearrangements occurred faster than in control oocytes indicating that overexpressing the active form of JNK accelerated egg apoptosis (Figure 5C). Altogether, these results clearly show that the apoptotic death of unfertilized oocytes depends on both JNK and Cdk1 activities.

### The proapoptotic factor Bad is controlled by JNK and mediates egg apoptosis in unfertilized eggs

**Figure 6:**
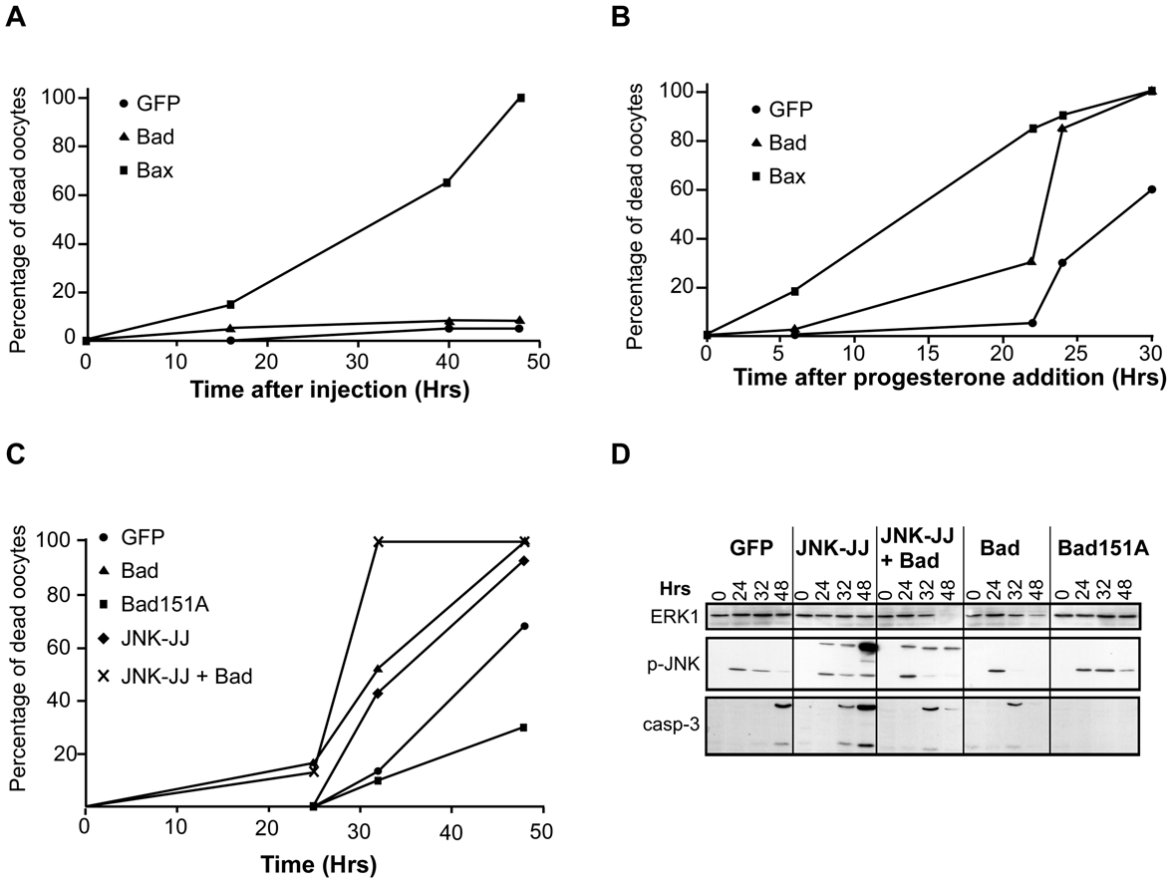
Bad and JNK control egg apoptosis in a cooperative manner. (A) and (B) Prophase oocytes were microinjected with mRNAs encoding either GFP (black circles), Bax (triangles) or Bad (squares) and were stimulated (B) or not (A) with progesterone one hour later. Egg death was monitored by external egg morphology at the indicated times. (C) and (D) Oocytes were microinjected with mRNAs encoding either GFP (white circles), the JNK-JJ (black diamonds), Bad (black triangles), a dominant negative form of Bad (BAD151A, black squares) or a mix of mRNA encoding for both JNK-JJ and Bad (crosses). One hour later, oocytes were incubated with progesterone (time 0). (C) Death time-course was monitored by egg morphological changes at the indicated times. (D) Western blot analysis of egg lysates using antibodies directed against ERK1, the active phosphorylated form of JNK (p-JNK) and active caspase 3 (casp-3). Note that the JNK-JJ form migrates slower than endogenous JNK. These data are representative experiments repeated on oocytes and eggs from three different females.

Several reports have revealed the role of JNK in regulating Bad phosphorylation [35]–[38]. This prompted us to investigate the possibility that Bad could be involved in the apoptosis of unfertilized eggs. Prophase-blocked oocytes were microinjected with mRNAs encoding either Bax or Bad. Overexpression of Bad did not promote apoptosis whereas Bax led to a complete cell death within 50 h (Figure 6A). This result shows that prophase oocytes, which do not spontaneously undergo apoptosis, are equipped with a mitochondrial death machinery that can be activated by overexpressing Bax but not Bad. Since oocytes spontaneously die only once they are matured, we examined whether the proapoptotic activity of ectopic Bad could depend on the completion of meiotic maturation. Oocytes were injected with mRNAs encoding either Bax or Bad, were then stimulated by progesterone and the spontaneous death affecting matured oocytes was examined. As expected, Bax strongly accelerated the death process (Figure 6B). Interestingly, overexpressed Bad that had no effects in prophase-arrested oocytes, also shortened the time of apoptosis induction of matured oocytes (Figure 6B). These data suggest that ectopic Bad is kept inactive in prophase oocytes and acquires its pro-apoptotic capacity during meiotic maturation.

To investigate whether endogenous Bad is involved in the apoptotic process affecting unfertilized eggs, we injected a mRNA encoding a dominant-negative form of Bad, Bad151A [39]. In contrast to the wild type form of Bad that accelerates the spontaneous egg death, Bad151A strongly delayed the process, as judged by external egg morphology and caspase 3 activation (Figure 6C and 6D). This result shows that endogenous Bad is actively involved in the apoptosis execution. To determine whether JNK and Bad collaborate in the control of the apoptosis of unfertilized eggs, mRNA encoding JNK-JJ, the constitutive active form of JNK, was injected in eggs in the presence or absence of Bad. JNK-JJ was expressed and activated in the oocyte, as detected by western blot, its electrophoretic migration being retarded in comparison to the endogenous JNK protein (Figure 6D). As already mentioned (Figure 5), overexpression of the active JNK-JJ form accelerated egg apoptosis (Figure 6C and 6D). Overexpression of both JNK-JJ and Bad produced a much stronger effect than the separate injection of each compound, leading to 100% of cell death within 30 h (Figure 6C and 6D). These results establish the positive cooperative roles played by JNK and Bad in the egg death process.

**Figure 7:**
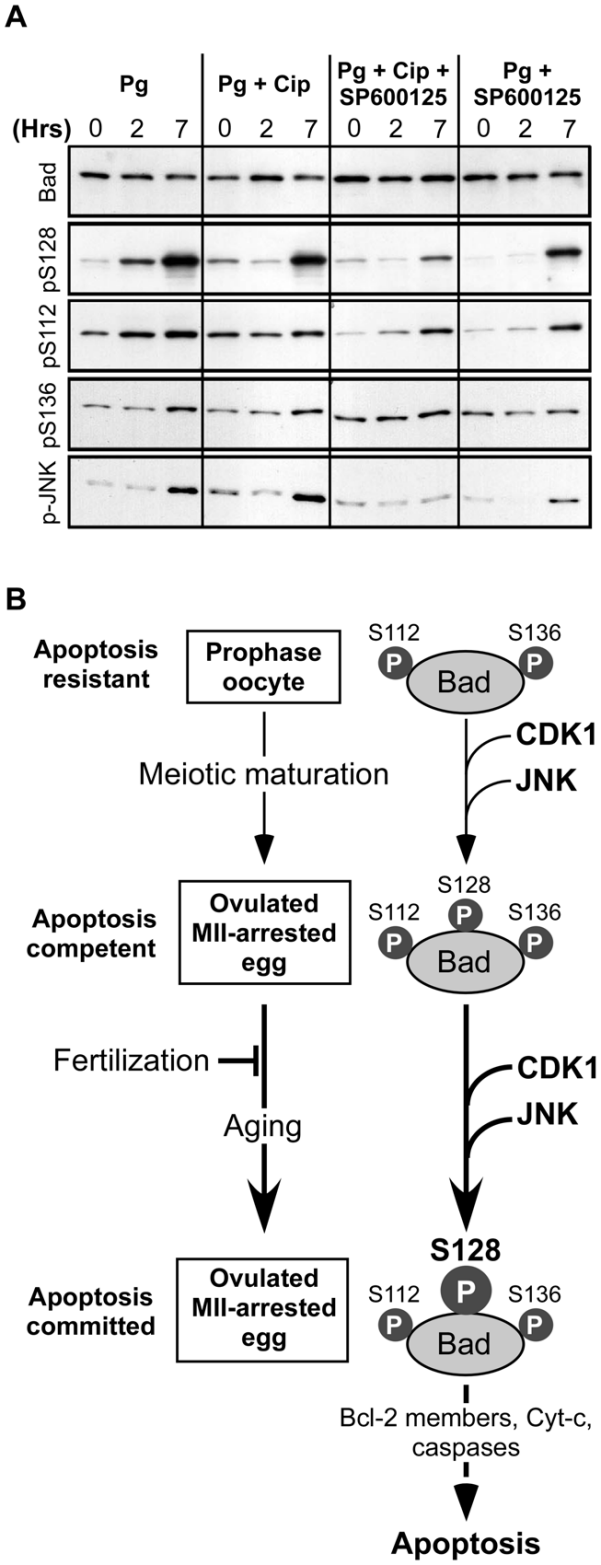
Egg apoptosis correlates with the phosphorylation of Ser128 of Bad. (A) Oocytes were microinjected with a mRNA encoding Bad-GFP and one hour later incubated in the presence or the absence of the JNK inhibitor, SP600125, or microinjected with p21^Cip1^ protein (Cip). They were then stimulated with progesterone (time 0). Egg proteins were analysed at the indicated times by immunoblot using antibodies against Bad, Ser128 (pS128), Ser112 (pS112) or Ser136 (pS136)-phosphorylated forms of Bad, and the active phosphorylated form of JNK (p-JNK). These data are representative experiments repeated on oocytes and eggs from at least three different females. (B) Regulation of apoptosis in unfertilized eggs. In the ovary, prophase oocytes are protected from apoptosis by an inhibited form of Bad phosphorylated at Ser112 and Ser136. At time of ovulation, the oocyte completes meiotic maturation. Bad becomes phosphorylated on Ser128 under the control of Cdk1 and JNK. During aging, the ovulated egg progressively accumulates increasing amounts of the Ser128 phosphorylated form of Bad that can ultimately trigger the death execution, unless fertilization occurs.

It has been reported that Bad is regulated by phosphorylation of several residues. The proapoptotic effects of Bad can be suppressed by phosphorylation of Ser136 and Ser112 that promote the interaction and sequestration of phosphorylated Bad by members of the 14-3-3 family of proteins [40]. In contrast, phosphorylation of Ser128 activates the apoptotic effect of Bad by counteracting its interaction with 14-3-3 proteins. Among kinases able to trigger phosphorylation of Ser128 of Bad are Cdk1 and JNK [37], [41]. We have shown that the egg apoptotic process depends on meiotic maturation, i.e. Cdk1 activation, and on JNK activity (Figures 2 and 5). Moreover, Bad activity is also dependent on meiosis resumption in eggs (Figure 6). These results prompted us to analyze Bad phosphorylation in the oocytes during meiotic maturation, by using antibodies recognizing specific phospho-residues of Bad. Since endogenous Bad was only weakly recognized by these antibodies, we expressed mammalian Bad as a tracer to analyze Bad phosphorylations. Bad mRNA was injected in prophase oocytes and progesterone was then added to promote meiotic maturation. Bad was expressed at a constant level and phosphorylated on Ser112 and Ser136 throughout the meiotic divisions (Figure 7A). In contrast, Bad was not phosphorylated on Ser128 in prophase-blocked oocytes and the phosphorylation of this residue took place during meiotic maturation (Figure 7A). To determine the contribution of Cdk1 and JNK to Bad phosphorylation, prophase oocytes overexpressing Bad were either injected with the p21^Cip1^ protein, or treated with the JNK inhibitor, SP600125. They were then stimulated by progesterone. As previously reported, p21^Cip1^ delayed meiotic maturation whereas SP600125 did not affect the process (not shown). Inhibition of Cdk1 did not significantly affect JNK activation but delayed the time-course of Ser128 phosphorylation of Bad (Figure 7A). SP600125 retarded and reduced both JNK activation and Ser128 phosphorylation of Bad whereas inhibition of both Cdk1 and JNK had cumulative effects on Bad phosphorylation (Figure 7A). Ser136 phosphorylation level of Bad was unaffected by inhibition of Cdk1 and/or JNK whereas Ser112 phosphorylation was slightly reduced by JNK inhibition (Figure 7A). We therefore concluded that the activating phosphorylation of Ser128 of Bad is promoted by Cdk1 and JNK during meiotic maturation whereas both phosphorylations of Ser112 and Ser136, which are known to inhibit Bad function, are not significantly regulated during this process.

## Discussion

This study demonstrates that the default fate of *Xenopus* ovulated oocytes is death by apoptosis. Prophase-blocked oocytes have a long life span. The oocyte growth period takes two years in the *Xenopus* ovary. The fully-grown oocytes then stand for several months in the ovary before ovulation. Our experiments show that prophase-blocked oocytes do not exhibit any sign of apoptosis when they are maintained *ex vivo* for days, in strong contrast with post-meiotic eggs. Clearly, although the oocytes possess all the apoptotic machinery, they will not engage spontaneously into the apoptotic process. Once the oocyte enters meiotic divisions, it acquires the competence to commit suicide and executes this death program if not fertilized. Meiotic maturation winds up a maternal molecular clock that ticks off the post-ovulation time towards death. If successful, the fertilization stops the clock. If not, the egg commits suicide.

### The *Xenopus* egg as a model system to study egg apoptosis

Apoptotic execution can be reproduced with *in vitro* extracts derived from *Xenopus* eggs obtained from females that have been hormonally primed to undergo pre-ovulatory death by a process analogous to follicular atresia [42], [43]. Therefore, this *in vitro* system does not provide an appropriate model system for studying ovulated egg apoptosis that occurs as a default fate if not fertilized. Our study introduces *Xenopus* eggs as an *in vivo* system for studying the death of unfertilized eggs. Since postovulatory eggs naturally age internally in mammals, it is of great importance to kill a potential parthenogenetic egg, able to generate ovarian teratoma. This observation is not restricted to viviparous species, as this study shows that a significant amount of ovulated eggs are recovered in the reproductive tract of the *Xenopus* female where they ultimately die by apoptosis. It is also tempting to speculate that eggs that fail to be fertilized need to be removed because they may suffer from aging and degeneration of maternal materials, leading to abortive or abnormal development [44], [45].

### Unfertilized eggs die by apoptosis

Using both morphological and molecular criteria, our study demonstrates that unfertilized egg death corresponds to apoptosis. Oocytes and eggs do not support transcription and we did not observe significant alteration of egg death in the presence of protein synthesis inhibitors (data not shown). Therefore, the egg death process occurs independently of transcription and translation. Although the morphological analysis of dying eggs reveals some findings reminiscent of apoptosis, as cell shrinking, the egg death process differs significantly from some canonical apoptotic features, such as membrane blebbing or cell fragmentation. At least two striking characteristics of the *Xenopus* egg probably account for this peculiar cell death, the size of this giant cell (1.2 mm) and the arrest in M-phase. Other animal cells commit apoptosis in interphase or in a differenciated state, starting with decondensed chromatin organized within the nucleus. Initiating apoptosis with condensed chromosomes organized in a spindle, obviously implies a specific morphological execution of cell death. The molecular analysis of *Xenopus* egg death unambiguously reveals that this process corresponds to apoptosis. Interestingly, even though the initiator caspases 2 and 8 are activated, they are recruited downstream of the mitochondrial phase and would retro-amplify the process. It was previously reported that nutrient depletion in *Xenopus* oocytes promotes apoptosis through activation of caspase 2 upstream of mitochondrial Cyt c release [46], [47], indicating that unfertilized eggs and nutrient-depleted oocytes die by different apoptotic pathways. Recently, it has been shown that the elevation of ornithine decarboxylase (ODC) activity during *Xenopus* oocyte maturation protects eggs from reactive oxygen species-induced apoptosis [48]. It would be interesting to determine whether the spontaneous apoptosis occuring during unfertilized egg aging is correlated with a loss of ODC activity.

### What molecular clock is set off during meiotic maturation to program cell death?

Although prophase oocytes express apoptotic proteins and respond to the overexpression of Bax by executing the death program, they do not spontaneously undergo apoptosis. The competence for apoptosis is acquired during the time of meiotic maturation, in parallel with the competence to be fertilized. The two master kinases orchestrating meiotic maturation, Cdk1-cyclin B and ERK1, are good candidates to participate in the apoptosis induction pathway.

The ability of ERK pathways to promote cell survival is well documented [49]. Experiments involving *in vitro Xenopus* eggs extracts have shown that extracts arrested in interphase are susceptible to an endogenous apoptotic program whereas extracts arrested in M-phase are not. The ERK1 pathway would be responsible for rendering the M-phase extracts refractory to apoptosis [50]. In contrast, it has been proposed that ERK activity is required for activation of pro-apoptotic signaling molecules in starfish unfertilized eggs [4], [51]. This surprising result could be related to a striking difference between *Xenopus* and starfish eggs, the former being arrested in M-phase while the others are arrested in interphase. In this *in vivo* study, we establish that ERK1 is not a central player in the *Xenopus* egg decision to commit suicide and that its inactivation is a downstream event depending on the execution of the mitochondrial phase of apoptosis.

The role of Cdk1 in modulating apoptosis is controversial [52]. Cdk1-cyclin B kinase is the inducer of oocyte meiotic maturation. Inhibition of Cdk1 activation in the *Xenopus* oocyte prevents meiotic maturation, as well as caspase activation and egg death, illustrating the requirement of Cdk1 activity for eggs to acquire the competence for apoptosis. Interestingly, we show that Cdk1 activity decreases during egg aging, shortly before initiation of cell death, and independently of this process. Whether or not this down-regulation is important for the death process remains to be investigated.

We analyzed the potential involvement of a third candidate, JNK, in the commitment to *Xenopus* egg death. The role of JNK in apoptosis is complex as it has been reported to have proapoptotic or antiapoptotic functions, depending mainly on cell type and death stimulus [30]. JNK activity increases abruptly during *Xenopus* oocyte meiotic maturation and stays active thereafter [31]. JNK would not be involved in meiotic maturation [33] but could exert a proapoptotic effect in mature oocytes [31]. Our results clearly establish that JNK plays a positive role in the induction of the signaling pathway leading to apoptosis in *Xenopus* unfertilized eggs.

We therefore demonstrate that Cdk1 and JNK are key players in launching the countdown that leads to egg death execution in the absence of fertilization. The most attractive targets for both Cdk1 and JNK are proteins of the Bcl-2 family. Many reports have proposed the implication of several of these proteins in the spontaneous apoptosis occurring in egg extracts, such as Bid, Mcl-1, BIR family members, Bcl-2 or Bax [24], [43], [53]–[55], although in most of these studies, exogenously added mammalian proteins have been employed. In contrast to these works, our study is conducted in whole eggs and proposes the pro-apoptotic protein Bad as a key regulator of apoptosis induction in unfertilized eggs. On stimulation by a death signal, Bad is regulated by phosphorylation and consequently translocates to the outer membrane of mitochondria, where it inactivates anti-apoptotic proteins and activates Bax, resulting in apoptotic cell death [14]. The combination of the phosphorylations of several Bad residues is critical in the balance between cell survival and cell death. Interestingly, it has been reported that Cdk1 and JNK can phosphorylate Bad at Ser128, thereby promoting its pro-apoptotic activity [37], [41], [56]. Altogether, our data demonstrate that Bad is essential for the spontaneous apoptosis of the *Xenopus* unfertilized eggs and is regulated during meiotic maturation by both JNK and Cdk1. In prophase oocytes, Bad is phosphorylated at Ser112 and Ser136, two phosphorylations known to inhibit its pro-apoptotic activity, and this phosphorylation level is almost unmodified during meiotic maturation. This indicates that Bad is efficiently prevented in promoting apoptosis in the prophase-blocked oocyte. Kinases and phosphatases that control Bad phosphorylation on these two inhibitory residues during the long period of oogenesis deserve investigation. At least three phosphatases, PP2A, PP1 and PP2C, have been proposed to function as gatekeeper for Bad-mediated apoptosis by targeting Ser112 and Ser136 [57]–[59], and interestingly, all three of them are involved in the control of meiosis resumption and egg activation [60]–[64]. In contrast, Ser128 of Bad is not phosphorylated in prophase-blocked oocytes and is then phosphorylated during meiotic maturation, under the control of both Cdk1 and JNK, accounting for the positive involvement of both kinases in the acquisition of the death competence of the egg. As illustrated in Figure 7B, we therefore propose that prophase oocytes that are kept healthy for very long periods in the ovary, are protected from apoptosis by an inhibited form of Bad, phosphorylated at Ser112 and Ser136. At ovulation, the oocyte completes meiotic maturation and Bad becomes phosphorylated on Ser128 under the control of Cdk1 and JNK. The ovulated egg is therefore equipped with a stockpile of Bad that contains inhibiting phosphorylations and increasing amounts of phosphorylated Ser128 that can ultimately trigger the death execution, unless fertilization occurs (Figure 7B). Further studies should continue to elucidate how fertilization ensures the embryo survival by suppressing the maternal apoptotic program.

## Materials and Methods

### Materials

*Xenopus laevis* females were purchased from Xenopus Express (France) and maintained under laboratory conditions. Reagents, unless otherwise specified, were from Sigma-Aldrich (Lyon, France). The protocol of animal handling and treatment was performed in accordance with the guidelines of the animal ethics committee of the Ministère de l′Agriculture of France and approved by the ethic committee. Permit were delivered by the Prefecture de Police de Paris (France), Department of Veterinary Services, Service de la Protection et Santé Animales et de la Protection de l′Environnement, in agreement the Code Rural and the 9-04-1988 arrêté. Permit numbers are as followed: animal facility agreement: number A75-13-17; authorization to manipulate animals: number 75–797; capacity certificate for using wild animals: number SA0401109.

### Preparation and handling of oocytes

*Xenopus* oocytes were prepared as described [65]. *Xenopus* eggs were obtained from females injected with 500 IU of human chorionic gonadotropin and dejellied with 2% cysteine hydrochloride (pH 7.8) before lysis. In some cases, oocytes were microinjected with 10 nl of 1 mg/ml mRNA or with 23 nl of 1 mM of the following caspase inhibitors: DEVD-FMK (caspase 3 specific, Promega), VDVAD-FMK (caspase 2 specific, Calbiochem) and IETD-FMK (caspase 8 specific, Calbiochem). Oocytes were incubated in the presence of 1 µM progesterone, 50 µM U0126 (Promega France), 100 µM SP600125 (Cell Signaling, Danvers, USA) or 100 µg/ml cycloheximide. Germinal Vesicle Breakdown (GVBD) was monitored by the appearance of a white spot at the animal pole. For molecular analysis, oocytes were lysed at 4°C in 10 µl of lysis buffer per oocyte (80 mM ß-glycerophosphate, pH 7.3, 20 mM EGTA, 15 mM MgCl_2_, 1 mM DTT), supplemented with a protease inhibitor mixture (Sigma, P8340). Lysates were centrifuged at 15,000 g, 4°C for 15 minutes, and supernatants frozen at −80°C. Western blot analysis and kinase activity assays were performed using the same lysate.

### Apoptosis assays

*Morphology* – The timing of cell death was assayed by looking at the morphological appearance of the eggs: disappearance of the white spot at the animal pole of the cell, shrinking of the pigmented area and contraction of the pigment in restricted areas.

*Caspase 3 and 9 cleavage* – Activation of caspase 3 and caspase 9 was measured by immunoblotting using antibodies that specifically recognize the cleaved activated form of each caspase.

*Protease activity assays* – Activities of caspases and calpain were assayed with 30 µl of egg extract (equivalent to 3 eggs) by fluorometric assay kits (Calbiochem for calpain and caspase 2, Promega for caspases 1 and 3, and Clontech for caspases 8 and 9) according to the manufacturer's instructions.

*Relocalisation of Bad, Bax and Cyt c* – To analyse Cyt c release, Bad or Bax relocalisation, eggs were lysed in 250 mM sucrose, 10 mM Hepes pH 7.5, 2.5 mM MgCl_2_, 1 mM DTT. Lysates were centrifuged first at 1000 g at 4°C for 10 minutes to remove lipids and vitellus, and then at 16,000 g for 30 minutes. The supernatant was considered as cytosolic fraction and the pellet as the organelle fraction [66]. Eggs were never frozen prior to lysis to prevent organelle breakdown.

### Western blotting and antibodies

Samples of 50 µg of proteins (equivalent to 0.25 to 2 oocytes or eggs) in Laemmli buffer [67] were electrophoresed on 10% or 15% SDS-PAGE (Amresco Inc., USA), transferred to nitrocellulose filters (Schleicher and Schuell) using a semi-dry blotting system (Millipore) and immunoblotted using the following antibodies: anti-*Xenopus* cyclin B2 [60], anti-phosphorylated ERK_1/2_ (New England Biolabs), anti-ERK1 and anti-*Xenopus* Mos (Santa Cruz Biotechnologies), anti-caspase 9 [23], anti-Cyt c, anti-Bax, anti-p150Glued (BD Bioscience, USA), anti-phosphorylated histone H2B (Upstate Biotechnology Inc., USA), anti-Ser128 phosphorylated Bad (Abcam, France), anti-caspase 3, anti-Ser10 phosphorylated histone H3, anti-phosphorylated JNK, anti-Bad, anti-Ser112 phosphorylated Bad and anti-Ser136 phosphorylated Bad (Cell Signaling, Danvers, USA).

The primary antibodies were detected with appropriated horseradish peroxidase-conjugated second antibodies (Jackson ImmunoReaserch laboratories) and the Western blot Chemoluminescence Renaissance kit (Perkin Elmer Life Sciences).

### Kinase assays

Cdk1 kinase activity was assayed in extracts (equivalent to 2 oocytes or eggs) in the presence of 0.5 mM PKI peptide, 1 µCi of [gamma^32^P]ATP (Dupont, NEN), 100 µM ATP and 0.2 mg/ml histone H1 (Boehringer) in 50 µl of kinase buffer (50 mM Tris-HCl, pH 7.2, 15 mM MgCl_2_, 5 mM EGTA, 1 mM DTT). Kinase reactions were stopped by adding Laemmli buffer [67] and boiling, followed by electrophoresis. After autoradiography, the bands corresponding to histone H1 were excised and the associated radioactivity was measured in a Wallac 1409 scintillation counter.

### Plasmids and mRNA synthesis

The mMESSAGE mMACHINE kit (Ambion) was used for *in vitro* mRNA transcription using the following plasmids, or a PCR product containing the T3 promoter, as templates: pcDNA3-Bax (a kind gift of Dr. L. Sachs, MNHN, Paris), pEBG-Bad plasmid (Cell Signalling), pEGFP-1 (Clontech), pCMV-GFP-XR11 [22], pJNK-JJ (a kind gift of Dr Carron, CNRS-UPMC, Paris), pEBG-Bad151A and pCMV-GFP-Mcl-1. The Quickchange mutagenesis kit (Stratagene) was used to introduce a point mutation in pEBG-Bad to obtain pEBG-Bad151A carrying a substitution of Leu151 with Ala in the BH3 amphipathic alpha-helix. To obtain pCMV-GFP-Mcl-1, *Xenopus laevis* Mcl-1 coding sequence was amplified from a oocyte cDNA library (Stratagene) by PCR using primers based on EST sequences (accession numbers BX85270 and CF290196). The PCR product was cloned in the TOPO TA vector (Invitrogen) and then subcloned into pEGFP-1 vector (Clontech). The coding sequence was submitted to Genebank (accession number FJ236836).
